# TNF-**α** Regulates Natriuretic Peptides and Aquaporins in Human Bronchial Epithelial Cells BEAS-2B

**DOI:** 10.1155/2013/159349

**Published:** 2013-11-28

**Authors:** Letizia Mezzasoma, Lucio Cagini, Cinzia Antognelli, Francesco Puma, Eugenio Pacifico, Vincenzo Nicola Talesa

**Affiliations:** ^1^Department of Experimental Medicine and Biochemical Sciences, University of Perugia, Polo Unico Sant'Andrea delle Fratte, 06156 Perugia, Italy; ^2^Thoracic Surgery Unit, Ospedale S. Maria della Misericordia, University of Perugia, S. Andrea delle Fratte, 06156 Perugia, Italy; ^3^Clinical Pathology and Hematology Unit, Ospedale S. Maria della Misericordia of Perugia, S. Andrea delle Fratte, 06156 Perugia, Italy

## Abstract

Postoperative-fluid retention is a severe complication frequently reported in patients undergoing major surgical procedures. The complex network of molecules involved in such a severe surgery-induced condition remains poorly understood. Inflammation has been proposed among the various causes of fluid retention. Since TNF-**α** is one of the main proinflammatory cytokine initially released after major surgery, it is reasonable to assume its involvement in fluid overload. Here, we showed that TNF-**α** selectively regulates key molecules involved in fluids balance, such as natriuretic peptides (NPs) and aquaporins, in human bronchial epithelial cells BEAS-2B. In particular, we found that TNF-**α** induced a decrease of arial natriuretic peptide, natriuretic peptide receptor-1, aquaporin-1 and aquaporin-5 and an increase of brain natriuretic peptide with a different involvement of nuclear factor-**κ**B and mitogen-activated protein kinases signaling pathway activation. Moreover, the observed changes in NPs expression, demonstrate inflammation as an additional cause of brain natriuretic peptide elevation, adding an important piece of information in the novel area of study regarding NPs and inflammation. Finally, we suggest that inflammation is one of the mechanisms of Aquaporin-1 and aquaporin-5 expression regulation. Therefore, in this exploratory study, we speculate that TNF-**α** might be involved in postoperative-fluid retention related to major surgery.

## 1. Introduction

Weight gain with edema formation is frequently reported in patients undergoing major surgical procedures, with an incidence as high as 40% [1]. Postoperative weight gain and fluid overload have been associated with poor survival [2] and complications [3, 4]. The causes of fluid retention are various, and not completely clear. One of them could be related to the systemic response induced by surgical stress and operative trauma, regulated by a complex network of endocrine, neuronal, and immunological mechanisms [5–7]. Such surgery-induced reaction leads to an early hyperinflammatory status that is essential for tissue repair and host defense [5]. Cytokines are thought to play a pivotal role in the pathogenesis of surgical trauma. They have local effects of mediating and maintaining the inflammatory response to tissue injury and also initiate some of the systemic changes which occur [8–10]. After major surgery, TNF-*α* is one of the main proinflammatory cytokines initially released in the damaged tissue where it stimulates the production and release of more cytokines, responsible for inducing the systemic changes known as the acute phase response [5, 10]. Therefore, it is reasonable to assume TNF-*α* involvement among the various causes of fluid retention and thus it would be very important to understand the mechanisms underlying its involvement in this area.

In a recent study, patients undergoing pulmonary lobectomy, showed a significant weight gain, correlated with fluid retention, and an early rise in the plasma concentrations of brain natriuretic peptide (BNP), a member of the natriuretic peptides (NPs) family [11]. In particular, a significant weight gain was found to be correlated with large volumes of fluids accumulation on the postoperative day 2, despite a negative intraoperative fluid balance and peroperative strict fluid restriction [11]. Moreover, the patients, none of whom developed signs or symptoms of heart failure during the postoperative period, showed, immediately after surgery (on day 1) a significant increase in BNP plasma concentration [11]. NPs are hormone/paracrine factors that are released by the heart in response to myocardial stretch and overload, modulating body fluid homeostasis [12, 13]. BNP, secreted from the cardiac ventricles, and atrial natriuretic peptide (ANP), secreted from the cardiac atria, activate the same transmembrane guanylyl cyclase-A/natriuretic peptide receptor-A (NPR-A or NPR-1) [14–17]. In addition to vasodilation, cardiovascular homeostasis, sodium excretion, and inhibition of aldosterone secretion, it is becoming increasingly recognized that NPs possess a much broader range of biological activities, including effects on endothelial function and inflammation [18, 19]. The genetic expression and secretion of ANP and BNP have been studied mainly in the context of cardiac diseases associated with neuroendocrine and hemodynamic changes [12, 13, 20]; however it has been pointed out that changes in BNP also occur in a context of an acute inflammatory process [19, 21–24].

Another family of proteins, aquaporins (AQPs), is deeply involved in the physiological response to change of fluid volume and osmolarity [25, 26]. AQPs are widely distributed in various tissues throughout the body and facilitate osmotically driven water transport across cell membranes [25–27]. Recently, AQPs involvement in edema development has been pointed out. In particular, it has been shown that AQP4 is an essential mediator in the formation and resorption of edema fluid from brain parenchyma [28] and that AQP1 and AQP5 might play an important role in lung edema [29]. In addition, AQP1 and AQP5 expression is decreased in lung inflammation [30, 31]. 

The aim of our work was to investigate the potential involvement of TNF-*α* in the regulation of ANP, BNP, and their receptor NPR-1, as well as AQP1 and AQP5, key molecules involved in body fluid homeostasis. In order to exclude any hemodynamic change able to modulate NPs expression, we carried out an *in vitro* study, in human bronchial epithelial cells BEAS-2B. 

## 2. Materials and Methods

### 2.1. Reagents

Human TNF-*α* was obtained from ImmunoTools GmbH (Friesoythe, Germany). The Nuclear Factor-*κ*B (NF-*κ*B) inhibitors, BAY 11–7082 and QNZ, as well as the p38 mitogen-activated protein kinases (p38 MAPK) inhibitor SB 203580, the c-Jun N-terminal kinases 1/2 (JNK 1/2) inhibitor SP 600125, and the extracellular-signal-regulated kinases 1/2 (ERK 1/2) inhibitor U-0126, were obtained from Santa Cruz Biotechnology, Inc. (Heidelberg, Germany) and were dissolved in dimethyl sulfoxide (DMSO). Human BNP was obtained from Phoenix Europe GmbH (Karlsruhe, Germany). Dexamethasone (DEX) was from Sigma-Aldrich (Milan, Italy). Rabbit polyclonal antibodies (Abs) against ANP, NPR-1, AQP1, and AQP5 as well as the mouse monoclonal Ab against *β*-actin and the appropriate HRP-conjugated secondary Abs, were purchased from Santa Cruz Biotechnology, Inc. (Heidelberg, Germany). Rabbit monoclonal Abs against Phospho-I*κ*B-*α* and Phospho-p38 MAPK were purchased from Cell Signaling Technology, Inc. (Danvers, MA).

### 2.2. Cell Culture and Drug Treatments

Human bronchial epithelial cell line BEAS-2B was purchased from American Type Culture Collection (ATCC) and was routinely maintained at 37°C in 5% CO_2_ in RPMI 1640, supplemented with 10% heat inactivated (1 h at 56°C) fetal calf serum, 1X Lglutamine, 1 mM sodium pyruvate, 1X nonessential amino acids, 100 units/mL of penicillin, and 0.1 mg/mL of streptomycin (Invitrogen, Milan, Italy). Forty-eight hours before study, cells were seeded onto six-well culture dishes at 300.000 cells/well. TNF-*α* was dissolved in distilled water and used at the concentrations of 10, 20, and 40 ng/mL for 24 h. Inhibitors, BAY 11-7082, QNZ, SB 203580, SP 600125, and U-0126 were dissolved in 0.5% DMSO. The anti-inflammatory glucocorticoid DEX was dissolved in 0.1% methanol. In independent experiments, BAY 11-7082, QNZ, SB 203580, SP 600125, and U-0126, and DEX were added to cells 60 min before TNF-*α* administration at the concentration of 1 *μ*M for BAY 11-7082 or DEX and 10 *μ*M for QNZ, SB 203580, SP 600125, or U-0126. DMSO or methanol final concentrations in each assay were 0.005% and 0.001%, respectively. Control cells with DMSO or methanol did not show any significant difference respect to control cells in RPMI 1640 medium; therefore all the relative treatments were compared to these latter controls.

### 2.3. Cell Viability

The effects of TNF-*α*, BAY 11-7082, QNZ, SB 203580, SP 600125, U-0126, DEX, and BNP treatments were measured with a standard trypan blue uptake assay. Cell cultures were also examined morphologically via light microscopy.

### 2.4. RNA Isolation and cDNA Synthesis

Total cellular RNA was isolated using TRIzol Reagent (Invitrogen, Milan, Italy) according to the manufacturer's instructions and 1 *μ*g was reverse transcribed using the RevertAid H Minus First Strand cDNA Synthesis Kit (Fermentas, Hanover, MD) and random primers System (Invitrogen, Milan, Italy), according to the manufacturer's instructions.

### 2.5. Quantitative Real Time SYBR Green PCR Analysis

We employed quantitative Real Time SYBR Green PCR analysis (qRT-PCR) on Mx3000P QPCR Systems (Agilent Technologies, Milan, Italy) to evaluate the expression of ANP (NM_006172), BNP (NM_002521), NPR-1 (NM_000906), AQP1(NM_198098), and AQP5 (NM_001651) versus ACTB (NM_001101). The sequences of oligonucleotide primers used for qRT-PCR and the thermal cycling conditions are summarized in Table 1. All primers were designed using Beacon Designer 4 software (Stratagene, La Jolla, CA), starting from published sequences data supplied by the NCBI database. Reactions were performed in a total volume of 25 *μ*L, containing 250 ng or 500 ng of cDNA, 1X Reagent Brilliant II SYBR Green QPCR Master Mix, and the appropriate concentration of the specific primers (Medical, Milan, Italy). Data for comparative analysis of gene expression were obtained using the ΔΔCt method, as described in the ABI Prism 7000 sequence detection system user guide [32]. Agarose gel electrophoretic analysis was used to check the predicted size amplicons for ANP (160 bp), BNP (148 bp), NPR-1 (151 bp), AQP1 (98 bp), and AQP5 (180 bp).

### 2.6. Western Blot Analysis

Total protein extracts (40 *μ*g) were separated by 12% sodium dodecyl sulfate-polyacrylamide gel electrophoresis (SDS-PAGE) and blotted onto a nitrocellulose membrane, using iBlot Dry Blotting System (Invitrogen, Milan, Italy). Nonspecific binding sites were blocked in Roti-Block (Roth GmbH, Karlsruhe, Germany) for 2 h at room temperature. The membranes were blotted overnight at 4°C with a 1 : 100 dilution of the primary specific Abs and washed with TBST. The antigen-Ab complex was detected by incubation of the membranes for 2 h at room temperature with the appropriated HRP-conjugated secondary Abs and revealed using the enhanced chemiluminescence (ECL) system by Amersham Pharmacia Biotech (Uppsala, Sweden).

As internal loading controls and for protein expression normalizing purpose, all membranes were subsequently stripped of the first antibody in a stripping buffer (100 mM 2-ME, 2% SDS, and 62.5 mM Tris-HCl, pH 6.8) for 30 min at 50°C followed by washings with TBST. The membranes were then reprobed with anti-*β*-actin antibody, followed by incubation with HRP-conjugated Ab. 

### 2.7. BNP Protein Concentration in Culture Medium

BNP protein concentration was measured in culture medium using the chemiluminescent enzyme immunoassay kit TRIAGE BNP (Biosite Incorporated, San Diego, USA). The minimum quantity of human BNP detectable with this system is 1.0 pg/mL. The intraassay and the interassay coefficients of variations were 3.1% and 4.5%, respectively. 

### 2.8. Statistical Analysis

Results were expressed as means ± SD of three independent experiments. The statistical significance of differences between treated and untreated cells was assessed by Student's *t*-test. Differences between groups were considered significant when *P* < 0.05.

## 3. Results

### 3.1. TNF-*α* Selectively Modulates ANP, BNP, and Their Receptor NPR-1 Expression in BEAS-2B Cells

**Figure 1:**
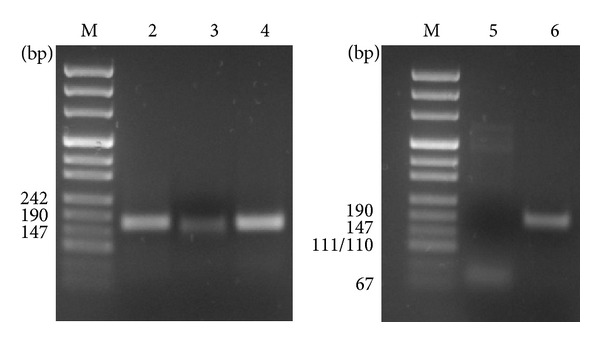
Identification of ANP, BNP, NPR-1, AQP1, and AQP5 transcripts in BEAS-2B cells. qReal-Time PCR was performed using gene specific primers for ANP (lane 2), BNP (lane 3), NPR-1 (lane 4), AQP1 (lane 5), sand AQP5 (lane 6) in BEAS-2B cells. Single bands of the predicted molecular size for ANP (160 bp), BNP (148 bp), NPR-1 (151 bp), AQP1 (98 bp), and AQP5 (180 bp) transcripts were detected. Lane M: DNA molecular marker.

**Figure 2:**
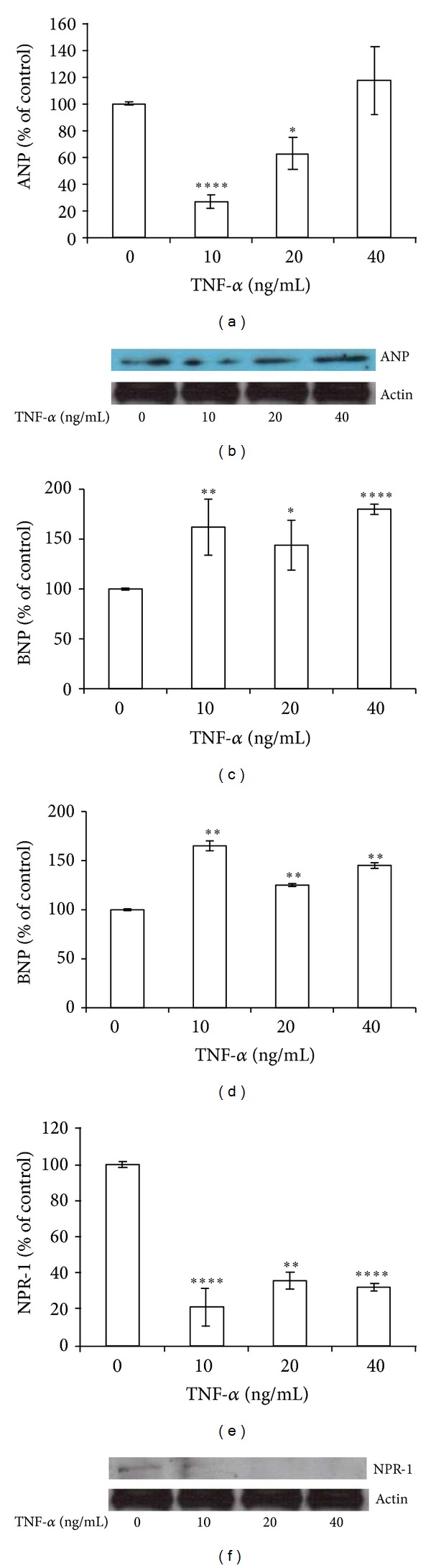
TNF-*α* selectively modulates ANP, BNP, and NPR-1 expression. BEAS-2B cells were treated with 10, 20, and 40 ng/mL TNF-*α* for 24 h. ((a), (b)) ANP, ((c), (d)) BNP, and ((e), (f)) NPR-1 gene expression at mRNA ((a), (c), and (e)) and protein level ((b), (d), and (f)). Western blots were obtained by using the specific rabbit polyclonal Abs. The blots were stripped of the bound Ab and reprobed with mouse anti-*β*-actin, to confirm equal loading. Western blots are representative of three separate experiments. All histograms indicate means ± SD of three different cultures each one tested in quadruplicate and expressed as percentage of control (*P < 0.05, **P < 0.01, ***P < 0.001, and ****P < 0.0001 versus untreated cells).

We firstly demonstrated that BEAS-2B cell line expresses ANP, BNP, NPR-1, AQP1, and AQP5. By qRT-PCR analysis, fragments of the predicted molecular size were generated (Figure 1). To determine whether the expression of ANP, BNP, and NPR-1 was affected by TNF-*α*, BEAS-2B cells were treated with 10, 20, or 40 ng/mL TNF-*α* for 24 h. ANP mRNA was significantly decreased, about 75% (*P* < 0.0001) and about 40% (*P* < 0.05) of control, after treatment with 10 and 20 ng/mL TNF-*α*, respectively. Forty ng/mL TNF-*α* induced an upregulation (about 20% of control) of ANP expression, even though not statistically significant (*P* > 0.05) (Figure 2(a)). A similar trend was also observed at protein level (Figure 2(b)). BNP mRNA expression was significantly (*P* < 0.01) increased (62% of control) since 10 ng/mL TNF-*α* treatment, maximum stimulation (80% of control) being obtained with 40 ng/mL (*P* < 0.0001) (Figure 2(c)). Treatment with TNF-*α*, at the above described concentrations, also increased BNP protein secretion in culture medium (*P* < 0.01) (Figure 2(d)). NPR-1 gene expression level was dramatically decreased (70–80% of control) after TNF-*α* exposure at all the used doses (*P* < 0.001) (Figure 2(e)), paralleled by a decrease of protein expression (Figure 2(f)).

### 3.2. NPs and NPR-1 Modulation by TNF-*α* May Require Activation of NF-*κ*B Signaling Pathway in BEAS-2B Cells

**Figure 3:**
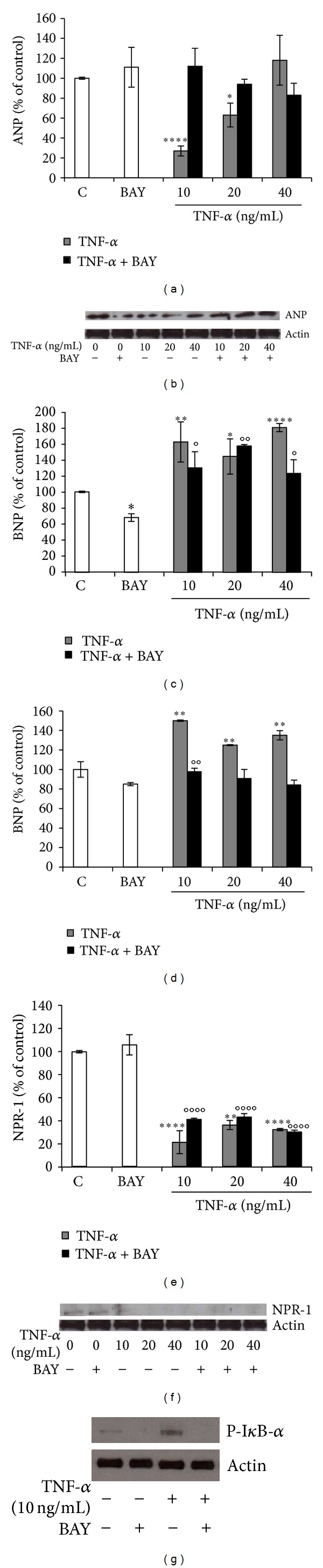
TNF-*α* modulation of NPs and NPR-1 may require activation of NF-*κ*B pathway. BEAS-2B cells were treated with 10, 20, and 40 ng/mL TNF-*α* alone or in combination with NF-*κ*B inhibitor BAY 11-7082 (1 *μ*M), for 24 h. ((a), (b)) ANP, ((c), (d)) BNP, ((e), (f)) NPR-1 gene expression at mRNA ((a), (c), and (e)) and protein level ((b), (d), (f)), and (g) Phospho-I*κ*B-*α* protein level. Western blots were obtained by using the specific rabbit polyclonal or monoclonal Abs. The blots were stripped of the bound Ab and reprobed with mouse anti-*β*-actin, to confirm equal loading. Western blots are representative of three separate experiments. All histograms indicate means ± SD of three different cultures each of one tested in quadruplicate and expressed as percentage of control (*P < 0.05, **P < 0.01, ****P < 0.0001 versus Controls). (°*P* < 0.05, °°*P* < 0.01, and °°°°*P* < 0.0001 versus BAY). C: Controls (untreated cells).

As known, TNF-*α* regulates numerous genes essential to the inflammatory process, through the activation of multiple signal transduction pathways, including NF-*κ*B [33]. Therefore, to investigate the possible involvement of NF-*κ*B signaling pathway in TNF-*α*-mediated regulation of NPs and NPR-1, we used the specific NF-kB inhibitor BAY 11-7082, that diminishes the activation of NF-*κ*B, by preventing phosphorylation of its inhibitory I*κ*B-*α* protein. As shown in Figure 3(a), TNF-*α*-induced downregulation of ANP was completely reverted by BAY 11-7082 at both mRNA and protein level (Figure 3(b)), suggesting that this response requires NF-*κ*B activation pathway. Regarding TNF-*α*-induced BNP upregulation, this was only partially reverted by BAY 11-7082 at mRNA level (Figure 3(c)), while was completely reverted at protein level (Figure 3(d)). Conversely, TNF-*α*-induced decrement in NPR-1 expression was not affected by BAY 11–7082 either at mRNA or protein level (Figures 3(e) and 3(f)).

**Figure 4:**
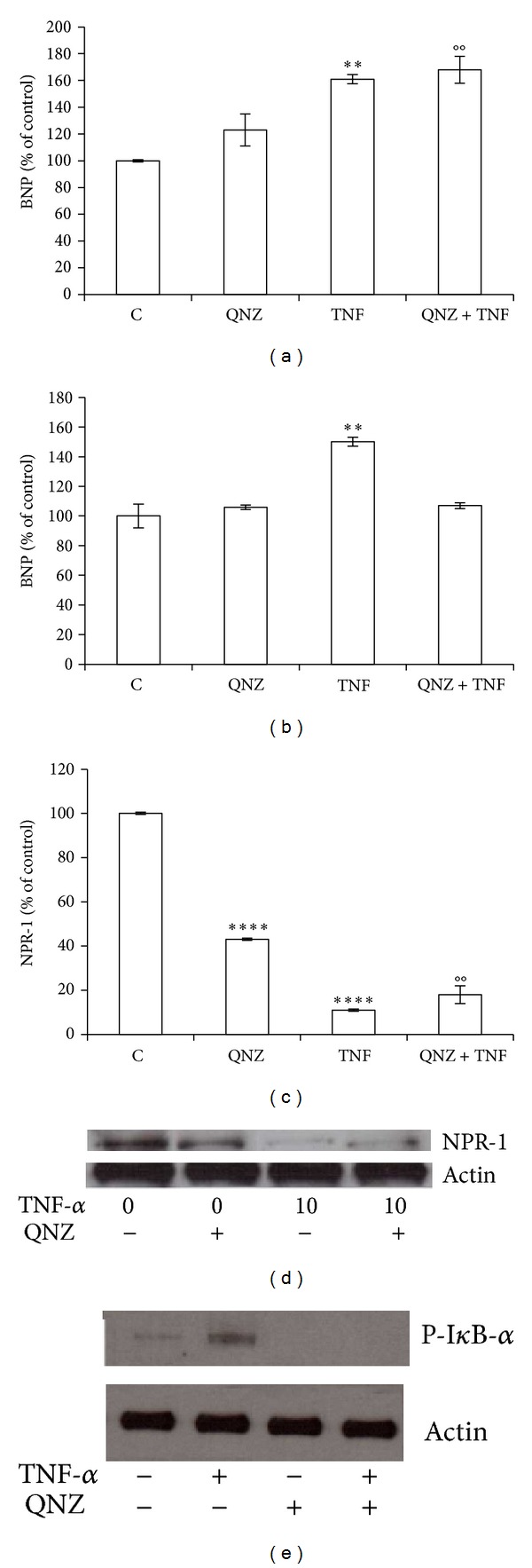
TNF-*α* modulation of protein levels of BNP and NPR-1 may require activation of NF-*κ*B pathway. BEAS-2B cells were treated 10 ng/mL TNF-*α* alone or in combination with the inhibitor QNZ (10 *μ*M), for 24 h. ((a), (b)) BNP, ((c), (d)) NPR-1 gene expression at mRNA ((a), (c)) and protein ((b), (d)) level, and (e) Phospho-I*κ*B-*α* protein level. Western blots were obtained by using the specific rabbit polyclonal or monoclonal Abs. The blots were stripped of the bound Ab and reprobed with mouse anti-*β*-actin, to confirm equal loading. Western blots are representative of three separate experiments. All histograms indicate means ± SD of three different cultures each one tested in quadruplicate and expressed as percentage of control (**P < 0.01, and ****P < 0.0001 versus Controls). (°°*P* < 0.01 versus QNZ). C: Controls (untreated cells).

Western blot analysis for the Ser^32^-phosphorylated I*κ*B-*α* protein proved the biochemistry evidence of the inhibitory action of BAY 11-7082 on NF-*κ*B activity (Figure 3(g)). To further investigate the role of NF-*κ*B in the modulation of BNP and NPR-1 in BEAS-2B cells after TNF-*α* treatment, we employed the NF-*κ*B-DNA binding inhibitor QNZ, which prevents free NF-*κ*B from binding to DNA [34]. The results obtained with such additional inhibitor, confirmed the previous findings obtained with BAY 11-7082. In particular, TNF-*α*-induced BNP up-regulation was not reverted by QNZ at mRNA level (Figure 4(a)), while it was completely reverted at protein level (Figure 4(b)). Accordingly, also TNF-*α*-induced decrement of NPR-1 expression was not affected by QNZ, either at mRNA or protein level (Figures 4(c) and 4(d)). Western blot analysis for the Ser^32^-phosphorylated I*κ*B-*α* protein proved the biochemistry evidence of the inhibitory action of QNZ on NF-*κ*B activity (Figure 4(e)).

### 3.3. BNP and NPR-1 Modulation by TNF-*α* May Require Activation of MAPKs Signaling Pathway in BEAS-2B Cells

**Figure 5:**
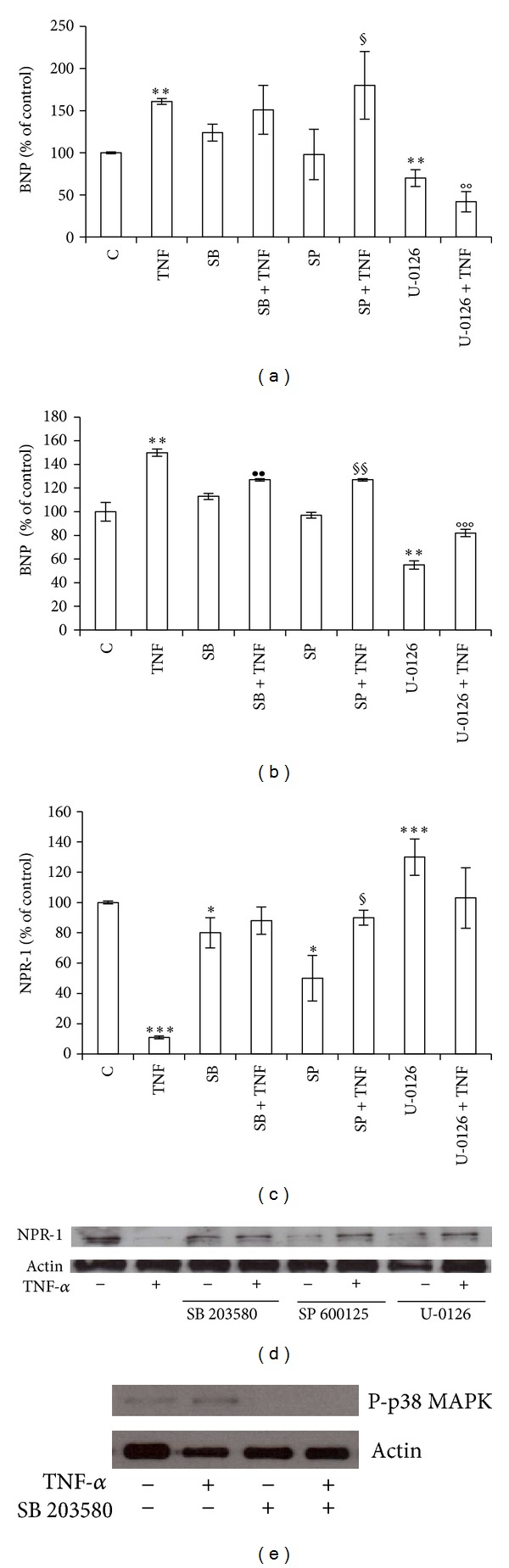
TNF-*α* modulation of BNP and NPR-1 expression may require activation of MAPKs pathway. BEAS-2B cells were treated with 10 ng/mL TNF-*α* alone or in combination with 10 *μ*M SB 203580 (p38 MAPK inhibitor), SP 600125 (JNK 1/2 inhibitor), or U-0126 (ERK 1/2 inhibitor), for 24 h. Panels show: ((a), (b)) BNP, ((c), (d)) NPR-1 gene expression at mRNA ((a), (c)) and protein ((b), (d)) level, and (e) Phospho-p38 MAPK protein level. Western blots were obtained by using the specific rabbit polyclonal or monoclonal Abs. The blots were stripped of the bound Ab and reprobed with mouse anti-*β*-actin, to confirm equal loading. Western blots are representative of three separate experiments. All histograms indicate means ± SD of three different cultures each one tested in quadruplicate and expressed as percentage of control. (*P < 0.05, **P < 0.01, and ***P < 0.001 versus Controls) (^••^ *P* < 0.01 versus SB) (^§^ *P* < 0.05,  ^§§^ *P* < 0.01 versus SP) (°°*P* < 0.01, °°°*P* < 0.001 versus U-0126). C: Controls (untreated cells).

Because TNF-*α* is able to activate multiple signal transduction pathways, including MAPKs, and because the activation of p38 MAPK and JNK participates in the regulation of inflammatory processes in bronchial epithelial cells [35, 36], we then investigated MAPKs pathway involvement in TNF-*α* modulation of BNP and NPR-1 expression. TNF-*α*-mediated up-regulation of BNP was not affected by SB 203580 or SP 600125, p38 MAPK, and JNK 1/2 inhibitors, respectively, while it was completely reverted by the ERK 1/2 inhibitor U-0126 (Figures 5(a) and 5(b)), suggesting that ERK 1/2 activation pathway is required for BNP TNF-*α*-mediated modulation. Regarding NPR-1, the TNF-*α*-mediated down-regulation was completely reverted by the use of all inhibitors (Figures 5(c) and 5(d)). Western blot analysis for the Thr^180^/Thr^182^-phosphorylated form of p-38 MAPK proved the biochemistry evidence of the inhibitory action of SB 203580 on p38 MAPK activity (Figure 5(e)). 

### 3.4. TNF-*α* Decreases AQP1 and AQP5 Expression in BEAS 2B Cells

**Figure 6:**
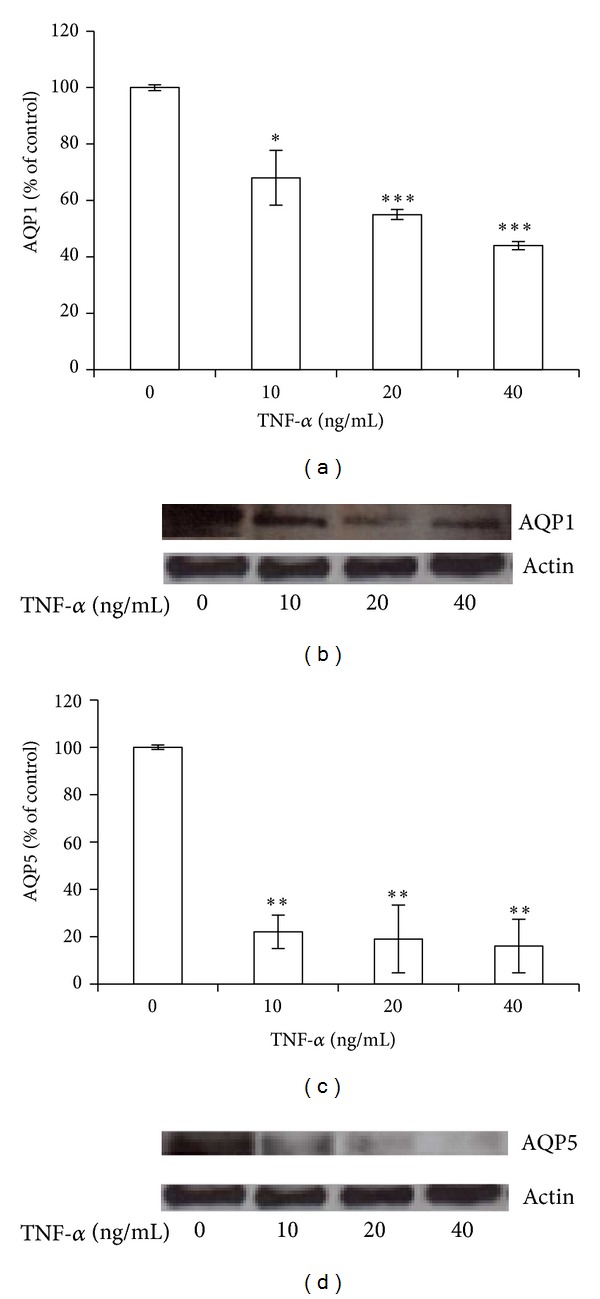
TNF-*α* decreases AQP1 and AQP5 expression in BEAS 2B cells. BEAS-2B cells were treated with 10, 20, and 40 ng/mL TNF-*α* for 24 h. Gene expression of AQP1 ((a), (b)) and AQP5 ((c), (d)) at mRNA ((a), (c)) and protein ((b), (d)) level. Western blots were obtained by using the specific rabbit polyclonal Abs. The blots were stripped of the bound Ab and reprobed with mouse anti-*β*-actin, to confirm equal loading. Western blots are representative of three separate experiments. All histograms indicate means ± SD of three different cultures each one tested in quadruplicate and expressed as percentage of control (*P < 0.01, **P < 0.001, and ***P < 0.0001 versus untreated cells).

We found that TNF-*α* induced a dose dependent down-regulation of AQP1 expression (*P* < 0.01) at mRNA (Figure 6(a)) and protein level (Figure 6(b)) and a dramatic decrease in AQP5 gene expression level (about 80% of control) with all the used doses (*P* < 0.001) (Figure 6(c)), paralleled by a comparable trend at protein level (Figure 6(d)). 

### 3.5. Decreased AQP1 and AQP5 Expression by TNF-*α* Does Not Require Activation of NF-*κ*B Signaling Pathway in BEAS-2B Cells

**Figure 7:**
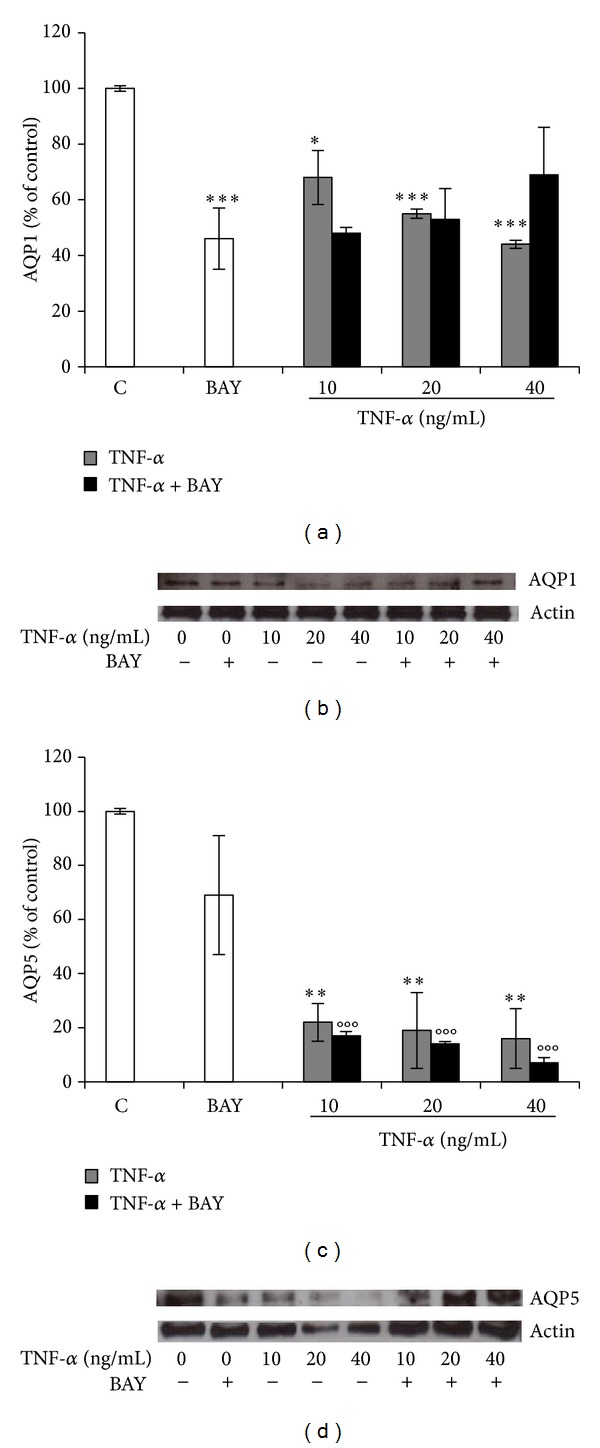
TNF-*α* downregulation of AQP1 and AQP5 expression may require activation of NF-*κ*B pathway.BEAS-2B cells were treated with 10, 20, and 40 ng/mL TNF-*α* alone or in combination with NF-*κ*B inhibitor BAY 11-7082 (1 *μ*M), for 24 h. Gene expression of AQP1 ((a), (b)) and AQP5 ((c), (d)) at mRNA ((a), (c)) and protein ((b), (d)) level. Western blots were obtained by using the specific rabbit polyclonal Abs. The blots were stripped of the bound Ab and reprobed with mouse anti-*β*-actin, to confirm equal loading. Western blots are representative of three separate experiments. All histograms indicate means ± SD of three different cultures each one tested in quadruplicate and expressed as percentage of control (*P < 0.01, **P < 0.001, ***P < 0.0001 versus Controls; and °°°*P* < 0.0001 versus BAY). C: Controls (untreated cells).

To examine a possible involvement of NF-*κ*B in TNF-*α*-mediated down-regulation of AQP1 and AQP5, BEAS-2B cells were incubated with BAY 11-7082. Cotreatment of such NF-*κ*B inhibitor with TNF-*α* resulted in a decrease of AQP1 expression, which was similar to that observed with TNF-*α* alone (Figures 7(a) and 7(b)), suggesting that the observed response does not require NF-*κ*B activation. Conversely, co-treatment of BAY 11-7082 with TNF-*α* affected AQP5 only at protein level (Figures 7(c) and 7(d)). 

### 3.6. BNP Effect on ANP, NPR-1, and AQP1 or AQP5 mRNA Expression in BEAS-2B Cells

**Figure 8:**
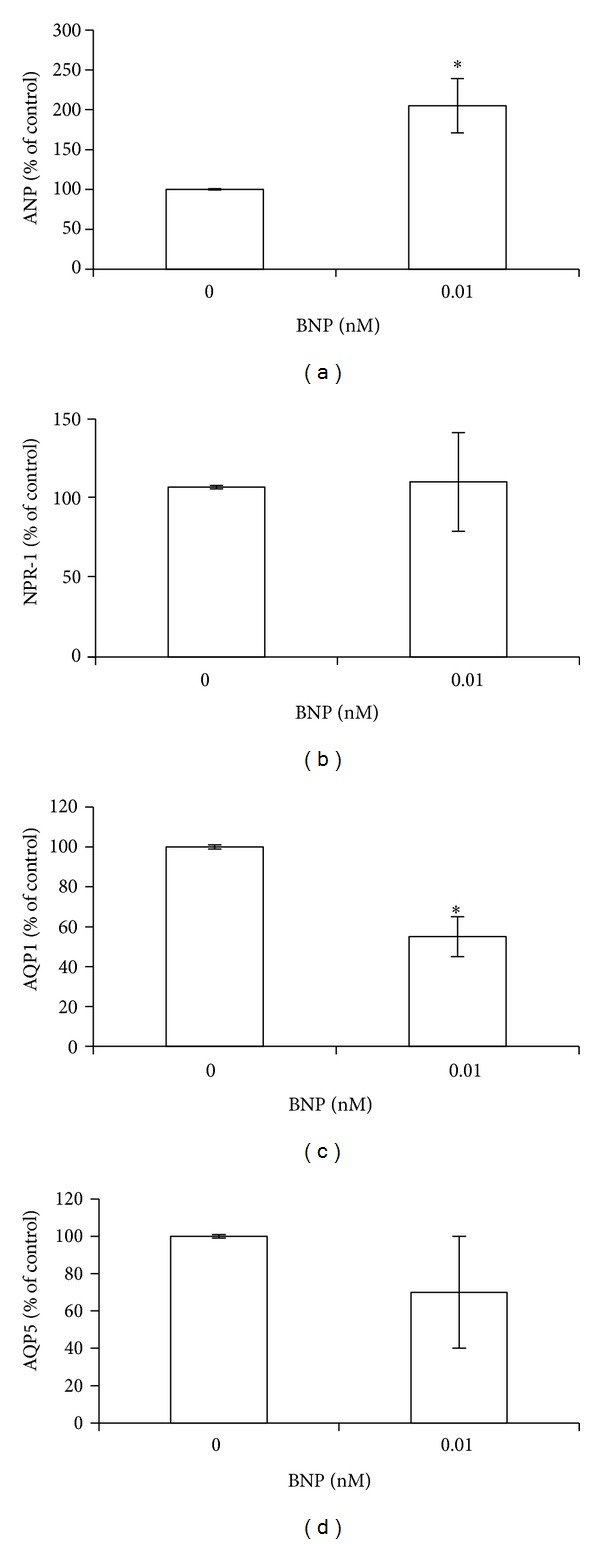
BNP effect on ANP, NPR-1, AQP1, and AQP5 expression.BEAS-2B cells were treated with 0.01 nM BNP for 24 h. Histograms show (a) ANP, (b) NPR-1, (c) AQP1, and (d) AQP5 gene expression at mRNA level. All histograms indicate means ± SD of three different cultures each one tested in quadruplicate and expressed as percentage of control (*P < 0.05 versus untreated cells).

In order to determine a possible direct involvement of BNP on the gene expression of ANP, NPR-1 and AQP1 or AQP5, BEAS-2B cells were treated with 0.01 nM BNP. This concentration was previously found to be accumulated in culture medium after 40 ng/mL TNF-*α* treatment, the dose that induced the maximum effect on the expression of the studied genes. BNP administration induced a significant (*P* < 0.05) increase (100% of control) on ANP mRNA level (Figure 8(a)) and did not affect NPR-1 expression (Figure 8(b)), while induced a marked decrease (45% of control) on AQP1 mRNA level (*P* < 0.05) (Figure 8(c)). A trend in decreasing was also observed for AQP5 gene expression, even though not statistically significant (*P* > 0.05) (Figure 8(d)).

### 3.7. Dexamethasone (DEX) Effect on BNP mRNA and Protein Levels in BEAS-2B Cells

**Figure 9:**
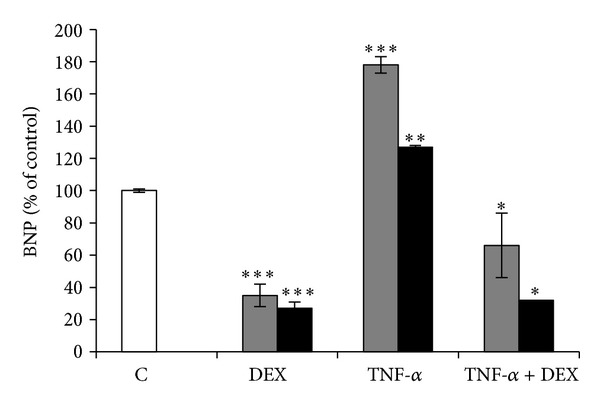
Dexamethasone effect on BNP expression. BEAS-2B cells were treated with 1 *μ*M dexamethasone (DEX) and 40 ng/mL TNF-*α* alone or in combination with DEX, for 24 h. Histograms show BNP gene expression at mRNA (gray bars) and protein (black bars) level. All histograms indicate means ± SD of three different cultures each one tested in quadruplicate and expressed as percentage of control (*P < 0.01, **P < 0.001, and ***P < 0.0001 versus Controls). C: Controls (untreated cells).

In order to study the capability of the anti-inflammatory drug DEX to affect TNF-*α*-mediated up-regulation of BNP mRNA and protein levels, BEAS-2B cells were treated with 1 *μ*M DEX. As shown in Figure 9, DEX induced a down-regulation of BNP expression and protein levels, alone or in combination with 40 ng/mL TNF-*α* (*P* < 0.01), demonstrating the capability of this glucocorticoid to revert TNF-*α* effects. 

## 4. Discussion

Postoperative-fluid retention is a severe complication frequently reported in patients undergoing major surgical procedures [1–4]. The complex network of molecules involved in such a severe surgery-induced condition remains poorly understood. After major surgery, TNF-*α* is one of the main proinflammatory cytokines initially released in the damaged tissue where it stimulates the production and release of more cytokines, responsible for inducing the systemic changes known as the acute phase response [5, 10]. Therefore, it is reasonable to assume TNF-*α* involvement among the various causes of fluid retention and very important to understand the mechanisms underlying its involvement in such an ambit. In the present study we demonstrated, for the first time to our knowledge, that TNF-*α* modulates the expression of ANP, BNP, NPR-1, AQP1, and AQP5 in human bronchial epithelial cells, BEAS-2B, via different involvement of NF-*κ*B and MAPKs signaling pathway activation. Here, we also provided the first demonstration that human bronchial epithelial cells express ANP, BNP, NPR-1, and AQP1 and that BNP is able to modulate AQP1 expression.

The observed expression of NPs and NPR-1 suggests an autocrine and/or paracrine function for these molecules and indicates important roles for NPs in several biological functions, including regulation of fluid movement across the airway epithelial cells, bronchial relaxation [37], vasodilatation, and pulmonary vascular permeability. About the former process, the role of ANP and BNP could be analogous to that hypothesized for NPs in transepithelial ion flux in the choroid plexus [38], in the kidney [39], colon [40], and retina [41].

The observed TNF-*α* capability of selectively regulating ANP, BNP, and NPR-1 gene expression in BEAS-2B cell line is of particular interest because it demonstrates that inflammation alters NPs expression levels. In particular, the significant up-regulation of BNP expression and protein secretion after TNF-*α* treatment shows that this modulation can occur independently of hemodynamic influences and that inflammation should be considered an extracardiac cause of BNP elevation. Besides, *in vitro* studies conducted in rat cardiomyocytes have shown that not only hemodynamic factors, but also neurohumoral factors, activated during heart failure, such as angiotensin II, endothelin and cytokines, cause BNP secretion [42–44]. In addition, in patients with cardiovascular diseases, plasma BNP levels have been shown to be also affected by low-grade inflammation [24] and a selective increase in BNP plasma levels has been proposed as a general feature of inflammation [19]. The lack on any apparent TNF-*α* dose-depending increase in BNP gene expression that we observed in our system could be due to an autocrine negative feedback action mediated by BNP itself, following binding to the NPR-1 receptor expressed in BEAS-2B cells.

The biological role of BNPup-regulation after TNF-*α* treatment remains to be elucidated. However, since it has been demonstrated that BNP regulates the production of major inflammatory molecules, such as IL-12, IL-10, leukotriene B, and prostaglandin E2 in human macrophages [45] and that BEAS-2B are able to release inflammatory molecules [46], it could be reasonable to assume that the observed BNP increase, after TNF-*α* treatment, may stimulate the production and release of such cytokines also from human epithelial bronchial cells. In addition, in *in vitro* studies, a potent BNP inhibitory action on the production of the glucocorticoid anti-inflammatory hormone cortisol has been described [47]. Therefore, BNP could be considered a proinflammatory molecule by inducing cytokines expression in target cells and inhibiting the production of anti-inflammatory hormones. Therefore, the observed BNP up-regulation after TNF-*α* treatment could concur in potentiating an inflammatory status. The increased production and release of specific cytokines, causing systemic changes known as the acute phase response [5, 8, 10], induced by TNF-*α*, may thus also occur via BNP up-regulation. 

Recently, a BNP rise has been observed in patients undergoing pulmonary lobectomy for lung cancer [11]. In particular, the patients, none of whom developed signs or symptoms of heart failure during the postoperative period, showed an early significant increase, on day 1, immediately after surgery, of the plasma concentrations of BNP which was followed on the postoperative day 2 by a significant weight gain correlated with large volumes of fluids accumulation, despite a negative intraoperative fluid balance and peroperative strict fluid restriction [11]. We suggest that such an increase could be, at least in part, explained by the ability of proinflammatory cytokines, such as TNF-*α*, to upregulate BNP gene expression and secretion, and that this up-regulation could enhance the major surgery-related early hyperinflammatory response, essential for tissue repair and host defense [5]. 

The observed dexamethasone capability to completely revert TNF-*α*-induced BNP up-regulation suggests that this anti-inflammatory glucocorticoid may prevent BNP-related effects. 

Regarding ANP, the observed significant down-regulation of mRNA and protein levels after 10, 20 ng/mL TNF-*α* treatment suggests that also this condition could affect local inflammation status. In fact, ANP administration determines anti-inflammatory effects in airway epithelial cells [48], playing important roles in modulating inflammatory response. In consideration of such ANP anti-inflammatory action, we speculate that the observed TNF-*α*-induced ANP down-regulation could concur in enhancing an inflammatory status. Differently from the above reported doses, 40 ng/mL TNF-*α* administration induced ANP up-regulation, even though not statistically significant. We suggest that this result might be due to a direct BNP involvement. In fact, a marked ANP up-regulation was observed after BNP treatment at the dose accumulated in culture medium after 40 ng/mL TNF-*α* treatment. 

The observed selective modulation in ANP and BNP gene expression after TNF-*α* treatment, in line with other studies in different cell models [19, 49, 50] could be related to a different regulation mechanism at transcriptional level, as suggested by our observation on the different involvement of NF-*κ*B and MAPKs signaling pathways. In particular, we demonstrated that ANP down-regulation, TNF-*α*-mediated, occurs via NF-*κ*B activation pathway, whereas BNP modulation requires ERK 1/2 activation pathway. NF-*κ*B involvement in TNF-*α*-induced ANP down-regulation is not opposed to the proinflammatory role of this transcription factor, being ANP a molecule that exerts an anti-inflammatory effect in airway epithelial cells [48]. Regarding the mechanism linking NF-*κ*B to the downregulation of ANP gene expression, we suggest that it could be similar to that recently proposed for Fibroblast Growth Factor 10 (FGF-10), where NF-*κ*B activation may lead to reduced gene expression, by recruiting inhibitory factors to specific gene promoters [51]. 

ANP and BNP biological functions occur through their binding to the same NPR-1 [13]. Here, we demonstrated, for the first time to our knowledge, TNF-*α* capability to downregulate NPR-1 mRNA and protein expression in BEAS-2B cells and that this regulation occurs via MAPKs signaling pathway activation. The consequence of the complex TNF-*α*-induced NPs and NPR-1 observed modulation, associated with their binding to the same receptor on BEAS-2B cells, makes it difficult to predict what would be the net resulting effect both *in vitro* and *in vivo*, in relation also to the presence of a possible autocrine regulation by NPs and/or to the effects of different cytokines activated by both BNP and TNF-*α*. We hypothesize that the TNF-*α*-induced downregulation of NPR-1, paralleled by an up-regulation of BNP, could also lead to a paracrine action of this peptide, possibly on the microvascular endothelium of the lung, where NPR-1 is densely expressed [18]. In this context, we speculate that the induction of local modification on BNP or ANP by TNF-*α* in human bronchial epithelial cells could alter the barrier permeability of the pulmonary microcirculation. Hence, TNF-*α*, acting on epithelial cells, could also indirectly contribute to endothelial cells barrier dysfunction and permeability, by enhancing the local production of proinflammatory BNP and by down-regulating the anti-inflammatory expression of ANP. Besides, exogenous synthetic ANP has been shown to protect from endothelial barrier dysfunction in *in vivo* and *in vitro* models of lung injury [18] and the therapeutical relevance of these experimental observations has been shown in intensive care patients without heart disease, where intravenous ANP infusion diminished pulmonary vascular permeability and pulmonary edema [18]. 

Regarding AQPs, we demonstrated that BEAS-2B cells express AQP1 and AQP5 and that TNF-*α* directly downregulates their expression at mRNA and protein levels. Regarding AQP1, also an additional BNP-mediated TNF-*α* involvement can be proposed. In fact, AQP1 down-regulation was observed with BNP treatment at the dose accumulated in culture medium after 40 ng/mL TNF-*α* treatment. The mechanisms that underlie AQPs regulation are of considerable interest. Several AQPs has recently been demonstrated to undergo complex regulation [27]. Our results show that AQP1 and APQ5 are subject to inflammation regulation, adding an important piece of information in this context. Our results are in agreement with other studies, describing a marked reduction in the expression levels of AQP1 and AQP5 in mouse lung [30] and mouse lung epithelial cells [31]. The biological significance of the observed TNF-*α*-induced AQP1 and AQP5 down-regulation is, at present, unclear. It has been recently suggested that the decrease in both AQPs may play an important role in the development of lung edema [52], which is known to be related with fluid retention. We, therefore, speculate that TNF-*α*-induced AQP1 and AQP5 down-regulation might play a role also in the excess of fluid accumulation, related to major surgical procedures.

## 5. Conclusions

In conclusion, our results provided evidence that, in human bronchial epithelial cells BEAS-2B, TNF-*α* selectively modulates mRNA and protein expression levels of different molecules involved in body fluid homeostasis: ANP, BNP, and their receptor NPR-1, AQP1, and AQP5. Moreover, our data pointed out that the changes, specifically affecting NPs expression, occur independently from hemodynamic influences and that inflammation should be considered an extracardiac cause of BNP elevation, adding an important piece of information in the novel area of study regarding NPs and inflammation. In addition, we demonstrated that such a modulation occurs via different involvement of NK-*κ*B and MAPKs signaling pathways. Finally, we suggested inflammation among the mechanisms involved in the regulation of AQP1 and AQP5. Therefore, in this exploratory study, we speculate that TNF-*α* might be involved in postoperative-fluid retention related to major surgery. 

## Figures and Tables

**Table 1:** Primer sequences, primer concentrations, and qRT-PCR cycling conditions.

Gene	Primer sequences	Primer concentrations	qRT-PCR cycling conditions
ANP	F: 5′-tcagcccagcccagagag-3′	200 nM	1 cycle at 95°C for 10 min; 40 cycles at 95°C for 1 min,
	R: 5′-gctccaatcctgtccatcctg-3′	200 nM	60°C for 30 sec and 72°C for 30 sec.

BNP	F: 5′-gagggcaggtgggaagcaaac-3′	200 nM	1 cycle at 95°C for 10 min, 40 cycles at 95°C for 1 min,
	R: 5′-gcaagaagagcaggagcaggag-3′	200 nM	60°C for 30 sec and 72°C for 30 sec.

NPR-1	F: 5′-ccctggaggtgctggctttgg-3′	200 nM	1 cycle at 95°C for 10 min; 40 cycles at 95°C for 1 min,
	R: 5′-ctctcaaggctactgggctcaacg-3′	200 nM	59°C for 30 sec and 72°C for 30 sec.

AQP1	F: 5′-ttggacacctcctggctattgact-3′	400 nM	1 cycle at 95°C for 10 min; 40 cycles at 95°C for 1 min,
	R: 5′-ccagtggttgctgaagttgtgtgt-3′	400 nM	60°C for 30 sec and 72°C for 30 sec.

AQP5	F: 5′-cgctcaacaacaacacaacgc-3′	200 nM	1 cycle at 95°C for 10 min; 40 cycles at 95°C for 1 min,
	R: 5′-ccagtgaagtagattccgacaagg-3′	200 nM	59°C for 30 sec and 72°C for 30 sec.

ACTB	F: 5′-cactcttccagccttccttcc-3′	600 nM	1 cycle at 95°C for 10 min; 40 cycles at 95°C for 1 min,
	R: 5′-acagcactgtgttggcgtac-3′	600 nM	59°C or 60°C for 30 sec and 72°C for 30 sec.

qRT-PCR: quantitative real time polymerase chain reaction; F: forward; R: reverse.
